# Measurement Reactivity to Hip-Worn Accelerometry Among Adolescent Females: A Concurrent Wear Approach

**DOI:** 10.1080/1091367x.2026.2704743

**Published:** 2026-07-16

**Authors:** Jacob Carson, Esmeralda Castro, Michael Higgins, Job Godino, Britta Larsen

**Affiliations:** a Herbert Wertheim School of Public Health and Human Longevity, University of California San Diego, La Jolla, California, USA;; b Family Medicine and Public Health, University of California San Diego, La Jolla, California, USA;; c EPARC, University of California San Diego, La Jolla, California, USA;; d Laura Rodriguez Research Institute, Family Health Centers of San Diego, San Diego, California, USA

**Keywords:** Physical activity, accelerometry, consumer wearables, pedometry, free-living activity

## Abstract

Device-based measurement of physical activity (PA) is crucial for valid research, but reactivity to these devices can undermine validity. Inconsistent findings in prior reactivity research warrant unique approaches among diverse populations, including those habituated to wearing another device. We aimed to estimate reactivity to ActiGraph GT3X+ via concurrent Fitbit Inspire HR wear among Latina adolescents using a person-day matched cross-sectional design. Fitbit data estimated steps, moderate-vigorous PA, light PA, and sedentary time for all days in the 2 weeks preceding and following matched ActiGraph wear days. Linear models estimated the association of ActiGraph wear day with activity after adjustment for covariates. There were 2987 total days across 107 participants, ~35% classified as ActiGraph wear days. Steps statistically significantly differed (*p =* .0499) with ActiGraph wear days corresponding to 248 greater average steps. Measurement reactivity to ActiGraph may impact steps among adolescent females. Future research could benefit from concurrent wear strategies if feasible.

## Introduction

Given the well-established, far-reaching benefits of physical activity (PA), developing effective strategies for promoting and measuring PA is key for population health (Sluijs et al., 2021). Gold-standard device-based measures, such as accelerometer devices, can quantify the impact of PA interventions and reduce measurement bias that is frequent with subjective measures, such as overreporting activity (Slootmaker et al., 2009). However, the use of devices can also introduce reactivity, in which participants alter their behavior as a product of knowing the behavior is being monitored.

Prior literature has identified short-term reactivity to worn devices such as accelerometers and pedometers among adults and children (Baumann et al., 2018; Dössegger et al., 2014; Ling et al., 2011). This reactivity threatens validity, as accelerometers are typically worn for 7–10 days only at key timepoints, but assumed to be representative of typical PA patterns. Identifying the extent of reactivity in different populations can inform the validity of device-based PA measures and evaluation of the effectiveness of behavioral interventions. This is especially important in developing interventions for low-activity populations, such as adolescent Latinas (Whitt-Glover et al., 2009). There are a growing number of interventions aimed at increasing the physical activity of adolescent Latinas to address inequitable activity levels, but the paucity of evidence on measurement validity among these groups undermines our ability to understand their efficacy (J. Carson et al., 2025; J. R. Carson et al., 2025; Castro, Carson, et al., 2026; Larsen et al., 2022). Therefore, research aimed at understanding the accuracy of and reactivity to device-based activity measures in Latina adolescents is necessary to address health equity.

Commercial wearable devices, such as Fitbits and Apple Watches, have rapidly grown in popularity. In the past decade, the number of wearable trackers that shipped worldwide grew by over one thousand percent (Ferguson et al., 2022). The 2025 American College of Sports Medicine annual report on the worldwide fitness trends identified wearable activity trackers as the top ranked trend, consistent with prior years (Newsome et al., 2024). As wearable activity trackers can support behavior change with real-time feedback, goal setting, and self-monitoring, numerous intervention studies have integrated these devices (Brickwood et al., 2019; Gal et al., 2018).

While wearables are typically used as intervention tools, they also offer a scalable, device-based approach to measuring PA in free-living conditions over long periods of time. Fitbits have shown acceptable reliability and validity for measuring moderate-to-vigorous physical activity (MVPA) and steps in free living conditions (J. Carson et al., 2025; Feehan et al., 2018). As commercial wearables are low burden and meant to be worn daily for long periods of time, wearers often habituate to them, thus they may offer more accurate representation of typical daily PA than high burden research-grade accelerometers worn for short time periods (Peng et al., 2021).

Previous research has typically focused on changes to activity over the course of short-term accelerometry wear as a measure of reactivity as opposed to leveraging multiple devices (König et al., 2022). In interventions using wearables for long-term wear and simultaneously introducing accelerometers at key timepoints, changes measured by wearables could also indicate reactivity to accelerometers. The *Chicas Fuertes* study, a fully powered clinical trial of a digital PA intervention for Latina adolescents included daily Fitbit wear throughout the year-long study, and also accelerometer wear at baseline, 6 months, and 12 months (Larsen et al., 2018). The purpose of the current analysis was to evaluate Fitbit-measured daily steps and MVPA immediately prior to and during accelerometer wear at 6 and 12 months to evaluate reactivity to accelerometers in Latina adolescents. The present analyses assessed reactivity to hip-worn ActiGraph accelerometers using a matched cross-sectional design by comparing Fitbit data at the day-level for each participant.

## Methods

### Study population

This study is a secondary analysis which used 6- and 12-month data from the Chicas Fuertes Randomized Controlled Trial, a mobile technology-based PA intervention for Latina adolescents based in San Diego, California (R01NR017876). Chicas Fuertes included 160 Latina Teens (ages 13–18) who participated in less than 150 min of MVPA per week and were not actively engaged in an exercise program at enrollment. The study received IRB approval (IRB # 182,070) in compliance with federal regulations (45 CFR §46.408). Eligible individuals of the parent study attended a virtual orientation to receive detailed information about the study. A research team member discussed the contents of the forms with the parent and teen, allowed them time to read them on their own, and answered any additional questions that arose. Interested individuals provided signed informed written assent and parental consent (for participants under 18) or signed consent (for 18-year-old participants). More detailed eligibility criteria, recruitment, CONSORT diagrams, and results for the parent study can be found in previously published works (J. R. Carson et al., 2025; Castro, Carson, et al., 2026; Larsen et al., 2022; Naqvi et al., 2024).

### Measures of physical activity

Objective PA was measured using two different validated measures. A hip-worn ActiGraph GT3X+ accelerometer captured PA for 7 days at 6 months, and again at 12 months, while the wrist-worn Fitbit Inspire HR was continuously worn throughout the course of the study (12 months), including the weeks when the ActiGraph was worn. While accelerometry was also captured at baseline, baseline visits were excluded given that the initial wear period was concurrent and any reactivity at baseline would be attributable to both devices, rather than the ActiGraph alone.

The ActiGraph GT3X+ is a triaxial accelerometer commonly worn on the hip that measures movement in order to identify activity intensity. It has been validated against heart rate telemetry and total energy expenditure in children (Janz, 1994; Melanson & Freedson, 1995; Trost et al., 2000). Accelerometers were delivered via mail with detailed instructions to wear the accelerometer on their left-side hip for an entire waking day (approximately 12 hours) for seven consecutive days. Valid wear time was defined as wearing the device for at least 600 min per day for 5 days or a total of at least 3000 min over 4 days, with wear time identified using the algorithm developed by Choi et al. (Choi et al., 2011).

The Fitbit Inspire HR uses a MEMS triaxial accelerometer and optical heart rate tracker that combine to determine PA intensity through a proprietary algorithm. Four potential levels of activity intensity are used: sedentary, light, moderate, and very active (Fitbit User Manuals-Fitbit Help Center, n.d.). This specific device has been validated in adults and older adults. Further, the full product range of Fitbit devices have been validated for measuring steps and PA intensity in hundreds of papers across all age groups, including adolescents (Fitabase-Research Device Data and Analytics, n.d.; Polhemus et al., 2023; Rodrigues et al., 2022). Participants were instructed to wear the Fitbit Inspire HR device continuously on their non-dominant wrist throughout the entire 12-month period, with regular text-message reminders to sync the device to ensure complete data capture. Previous work on the agreement in measuring MVPA between these two devices in the current sample have been published elsewhere, suggesting moderate agreement with greater differences at higher levels of activity (J. Carson et al., 2025).

### Data processing and alignment

To test reactivity to the ActiGraph, we identified all wear days for participants at both 6-and-12-month visits. For the purposes of these reactivity analyses, a valid ActiGraph day was considered to be any day with >600 min of wear-time, excluding days in the mail by excluding days with average counts per minute that were below thresholds indicating sedentary wear. ActiGraph wear time was validated by the Exercise and Physical Activity Research Center (EPARC - The Cutting Edge of Exercise Science [EPARC], n.d.).

For the Fitbit Inspire HR, a valid day was defined as having >600 min of heart-rate data (in alignment with ActiGraph and previous research in children) or >6000 steps in a day as to not erroneously exclude Fitbit observations with valid PA intensity data (Wing et al., 2022). This approach is consistent with previous approaches to exclude step counts that could be erroneous and to not exclude valid wear days that did not include heart-rate data (e.g., if the device was worn over a sleeve) (Brewer et al., 2017; EPARC, n.d.; Hardcastle et al., 2020; Larsen et al., 2020). Complete Fitbit data for all participants over the entire study period was downloaded from Fitabase, an online data-management platform for Fitbit devices (Small Steps Lab, LLC, San Diego, CA, USA). Given that ActiGraphs were only worn for discrete periods (7–14 days dependent on expedience of participant compliance) while Fitbit data were available continuously throughout the entire study period, Fitbit data were matched by date to the existing ActiGraph data. Day-level data were created by summing the heart-rate minutes for every valid day, matched by participant ID and date.

A participant was included in the primary analyses if they had valid data on both devices for any given day (i.e., criteria met for ActiGraph and Fitbit described above). The final analyses excluded any observations without any data for the 14 days preceding the first valid ActiGraph day through the 14 days after the last valid ActiGraph day for a given participant. This threshold was chosen to capture the average activity levels surrounding ActiGraph wear. To understand potential reactivity across all types of activity, analyses were conducted to compare steps, MVPA, Light Physical Activity (LPA), and sedentary time across ActiGraph wear and non-wear periods.

### Statistical analyses

All statistical analyses were completed in R version 4.4.0 (24 April 2024) and RStudio version 2025.09.2 + 418. Fitbit data were cleaned and reduced to the valid data as described above using the *dplyr*, *tidyverse*, and *lubridate* packages. Histograms and Cullen and Frey Graphs were used to assess the distributions and normality of the activities of interest and choose the appropriate statistical modeling distributions. This included negative binomial and negative binomials with quadratic variance to account for the skewed or heavily zero-weighted distributions common in physical activity measures. Main analyses were conducted using the *glmmTMB* package given its flexibility for different linear modeling and outcome distributions. Generalized linear models with mixed effects were used for all analyses, with the main outcome variable being each type of activity. In addition to ActiGraph wear day, additional covariates were included in the model to account for confounding. These variables included the number of valid heart-rate minutes (scaled for comparability with other continuous variables), measurement timepoint (i.e., six or 12 months), weekday or weekend, age, group assignment, and a dummy variable, created to compare each participant to their own observations (clustering for each individual modeled as a random intercept for each participant ID). This analytic approach leverages within-person comparisons, reducing confounding by stable individual characteristics. Significant findings were interpreted at the alpha = .05 significance level and *emmeans* and *ggplot* were used to calculate and plot the differences between ActiGraph wear and non-wear days to visualize any statistically significant differences. The *DHARMa* package was used to assess model fit and identify misspecifications before interpreting the results. Outliers were examined for plausibility of values and included in all analyses.

## Results

### Descriptive statistics

One hundred seven participants met the criteria for inclusion in the present analyses (average age of 15.8 years, SD = 1.67), totaling in 2987 observations (an average of 27.9 days per participant. SD = 14.56). Of the included days, 1056 (35.4%) were concurrent ActiGraph wear days. Participants had an average valid Fitbit wear time of 1210 (SD = 267) minutes per day with a mean 7430 (SD = 4200) daily steps, 29.3 (SD = 45) minutes of MVPA, 239 (SD = 95.2) minutes of LPA, and 1170 (SD = 113) minutes of sedentary time. The sample data primarily included weekdays as opposed to weekends (72.7% vs 27.3%, respectively) and the majority of data were from the 6-month timepoint (64.8%). In this current sample, 47.7% were randomized to the intervention condition in the parent study. Descriptive statistics by ActiGraph wear status are depicted in Table 1. Main findings for each outcome are depicted in Table 2.

### Regression model fit

Residual diagnostics from the DHARMa package across models indicated minor but statistically detectable deviations from assumptions, with no strong visual evidence of systematic lack of fit for any of the outcomes of interest.

### Daily steps

**Figure 1.**
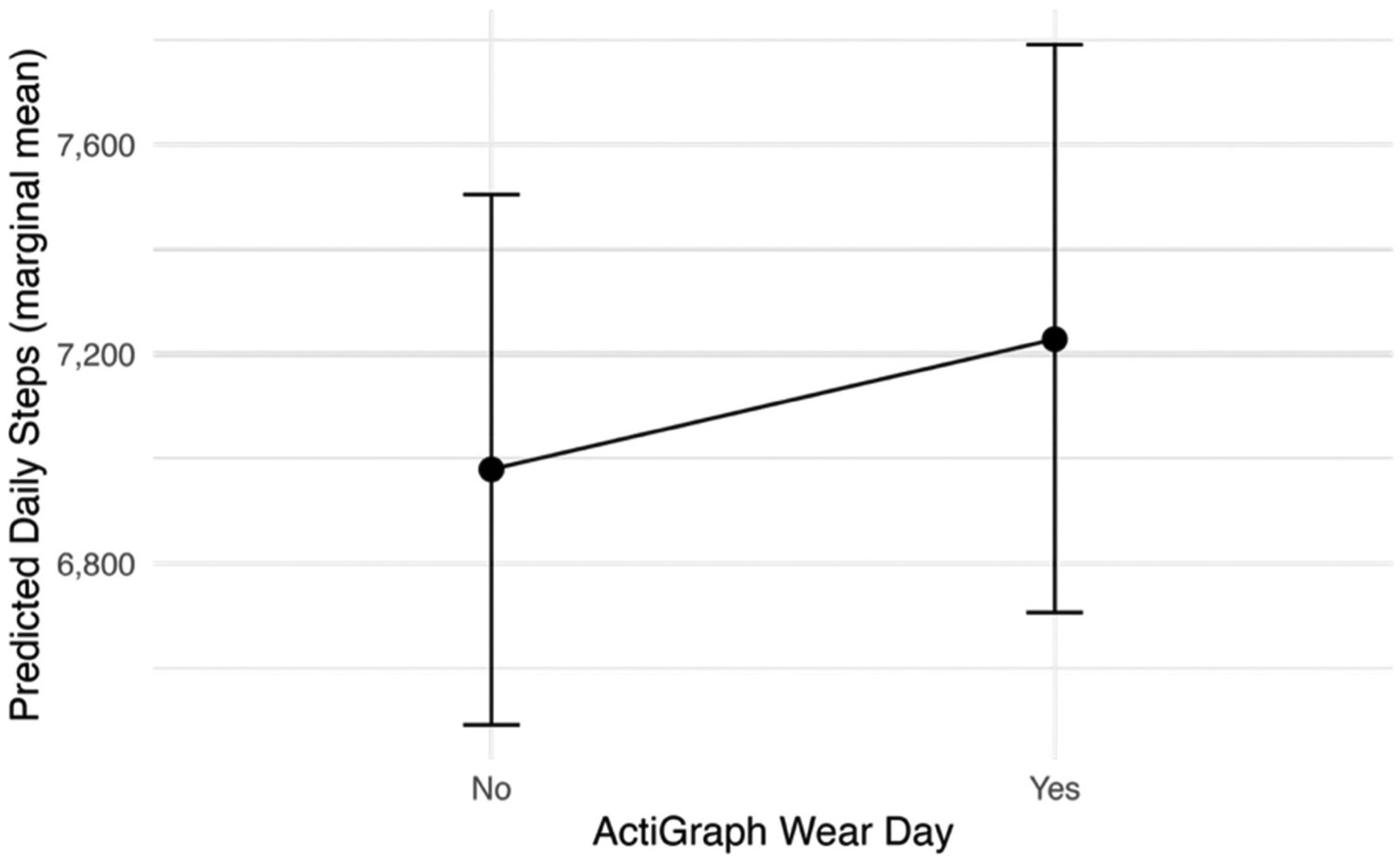
Predicted daily steps by ActiGraph wear day.

Across all monitoring days, the average within-participant standard deviation in daily Fitbit-measured steps was 3,339 steps/day. Given the distribution of daily steps, a negative binomial family was used to fit the linear model. In the fully adjusted model, days with ActiGraph wear had significantly higher average daily steps compared to non-wear days (Estimate = 0.03, 95% CI=(0.000, 0.070), *p* = .0499), as did the number of valid wear minutes (Estimate = 0.16, 95% CI=(0.102, 0.185), *p* < .001) while the 6-month timepoint (Estimate = −0.07, 95% CI=(−0.109, −0.030), *p* < .001) and weekends (Estimate = −0.17, 95% CI=(−0.208, −0.131), *p*=<.001) were associated with fewer daily steps. The the mean absolute difference based on ActiGraph wear is depicted in Figure 1, resulting in an average of 6980 (95% CI: 6492, 7504) vs 7228 (95% CI: 6706, 7791) for non-wear vs wear, respectively.

### Daily minutes of MVPA

Given the distribution of daily MVPA minutes a negative binomial family was used to fit the linear model. In the fully adjusted model, days with ActiGraph wear did not result in significantly different average daily MVPA compared to non-wear days (Estimate = 0.01, 95% CI= (−0.065, 0.086), *p* = .78). Only weekends were significantly associated with higher MVPA (vs. weekdays; Estimate = 0.10, 95% CI=(0.017, 0.191), *p* = .02).

### Daily minutes of LPA

Given the distribution of LPA, a gaussian family was used to fit the linear model. In the fully adjusted model, days with ActiGraph wear did not result in significantly different average daily LPA compared to non-wear days (Estimate = 4.01, 95% CI=(−1.860, 9.872), *p* = .18). The number of valid minutes was significantly associated with higher LPA (Estimate = 31.88, 95% CI=(28.280, 35.472), *p* < .001) while the 6-month timepoint was associated with lower LPA compared to the 12-month timepoint (Estimate = −13.79, 95% CI=(−20.302, −7.274), *p* < .001).

### Daily minutes of sedentary time

Given the distribution of sedentary time, a gaussian family was used to fit the linear model. In the fully adjusted model, days with ActiGraph wear did not result in significantly different average daily sedentary minutes compared to non-wear days (Estimate = −5.25, 95% CI=(−12.634, 2.127), *p* = .16). The number of valid minutes was significantly associated with lower sedentary time (Estimate = −31.24, 95% CI=(−35.739, −26.733), *p* < .001) while the 6-month timepoint was associated with higher sedentary compared to the 12-month timepoint (Estimate = 17.66, 95% CI=(9.480, 25.839), *p* < .001).

## Discussion

This study investigated the differences in Fitbit-measured PA between wear and non-wear days of hip-worn ActiGraph accelerometry among a sample of largely inactive adolescent Latinas with concurrent Fitbit wear time. ActiGraph wear was associated with a significant increase of ~ 250 steps per day on average, but was not significantly associated with MVPA, LPA, or sedentary time. It is worth noting that the statistical significance based on α = 0.05 is in itself arbitrary, but the difference in step estimates found here may have meaningful consequences for physical activity researchers. In this sample, the average daily step count on non-ActiGraph days (6,980 steps/day) fell just below the 7,000-step benchmark proposed by Paluch et al., whereas the average on ActiGraph wear days (7,228 steps/day) exceeded that benchmark (Paluch et al., 2021). Although this does not imply that individual participants meaningfully changed their activity classification, it illustrates that even modest differences attributable to reactivity could influence how study findings are interpreted. Acknowledging the role of reactivity in the determination of a marginally “significant” difference could help to better contextualize intervention research. Additional work should be done to understand what level of reactivity is meaningful for different research settings, including estimates of daily activity variability, of which only limited evidence exists (Castro, Farley, et al., 2026).

The non-significant findings for all other measures of activity could have multiple explanations that warrant further investigation. One explanation could be that reactivity to the ActiGraph manifests in specific types of activity, consistent with Baumann et al. (2018) which found reactivity in sedentary behavior and LPA but not MVPA (Baumann et al., 2018). Alternatively, these non-significant findings could be the result of measurement variability in the Fitbit. Previous research has found it to be more reliable and valid for steps compared to other types of PA, potentially resulting in reduced statistical power for non-step tests, which could explain the fewer significant estimates for all covariates in those models (Feehan et al., 2018). Finally, the non-significant findings could imply that reactivity does not extend beyond steps, providing additional confidence in the reliability and validity of the week-long ActiGraph estimates that are often used to objectively evaluate PA interventions (particularly those that focus on MVPA).

This study uniquely isolates reactivity to research-grade monitoring among participants already habituated to continuous wearable tracking, and our findings are consistent with the prior body of literature on ActiGraph reactivity. Among adults in a week-long study, light-intensity PA decreased over time while sedentary time increased (Baumann et al., 2018). One week-long study among Swiss children and adolescents identified that five percent of the PA results (measured in cpm) were attributable to reactivity (Dössegger et al., 2014). Participants were significantly more active on the first day than days two through six, but not significantly different from the final day of wear (Dössegger et al., 2014). The attenuation of the reactivity is shared by other research. Zhu et al. conducted a week-long study but among youth with moderate to severe intellectual disability, finding significantly higher light and moderate PA on the first wear day as opposed to days two through six, but significantly lower light PA on day seven (Zhu et al., 2020). A recent systematic review and meta-analysis examining reactivity from digital wearables found reactivity in ten of the fifteen included studies, specifically with increased steps and walking as compared to other kinds of PA, consistent with our findings (König et al., 2022). Overall, there were small to medium effect sizes for reactivity. Included studies were of short duration, typically 1 week, and none utilized a Fitbit to measure the reactivity (Diaz et al., 2015). In combination with this previous research, evidence of reactivity seems to be primarily a concern for non-MVPA measures, but still presents with small effect sizes. Given that the average within-subject standard deviation of daily steps was ~3339 steps, our observed mean absolute difference of 248 daily steps is modest, and therefore the practical significance is uncertain and context dependent. Unfortunately, outside of validation for accelerometry, information on population-level daily step variability among adolescents is limited, undermining our ability to contextualize this finding within the broader population. This limitation also underscores the value of continuous activity monitoring. Because physical activity naturally fluctuates over time, brief episodic accelerometer assessments may not fully capture habitual activity patterns, whereas longer-term wearable monitoring can help characterize these fluctuations and identify potential reactivity during accelerometer wear periods (J. Carson et al., 2025).

This study is strong in its relatively large sample size, evaluation of reactivity in a free-living sample, and addition to the literature by looking at reactivity in the demographic group with the lowest-levels of PA, thereby providing important information for the promotion of PA equity (Troiano et al., 2008; Whitt-Glover et al., 2009; Wolf et al., 1993). The unique contribution of our research is the use of concurrent Fitbit wear to measure differences in activity during ActiGraph wear and non-wear periods, adjusting for within- and between-subject variation to provide estimates specific to differences in device-wear. While this is a novel contribution, it simultaneously has limitations. As reactivity may depend on the target behavior and can be short-lived, such as a couple of days within start of wear, our analysis is limited in evaluating how reactivity persists for longer durations of time or for behaviors that are potentially less reactive, such as moderate and vigorous PA (König et al., 2022). We are also unable to determine any long-term reactivity to the Fitbit itself or other self-monitoring; however, the inclusion of and adjustment for multiple timepoints of data may partially attenuate those biases and the estimates still represent differences in activity due to ActiGraph wear specifically. Reasons for initial reactivity could be due to increased attention to a behavior, receiving feedback from a monitoring device, or even the excitement of having a new device. Additionally, while previous literature has looked at how the effect of reactivity diminishes over time, limitations of relying on valid concurrent wear precluded us from exploring this angle of research. Similarly, we were only able to compare the wear period to the period immediately before, not immediately after ActiGraph wear, due to limited data. Additionally, the strong association with valid wear time suggests that differences in measurement completeness may partially contribute to observed effects, as days with higher device wear may capture more activity. Last, due to the secondary analysis from a preexisting randomized controlled trial, these findings are limited to a largely inactive sample of Latina adolescents who increased their activity levels over time, which, although providing useful evidence for a key demographic group, limits generalizability to other populations despite the limited exclusion criteria and high retention of the parent trial (Naqvi et al., 2024).

This study is novel in its approach to measuring reactivity and presents the value of utilizing concurrent measurement in PA interventions and observational studies where possible. In addition to the present analysis, the dataset derived from the Chicas Fuertes RCT has allowed for other measurement comparisons that are unique to concurrent wear (J. Carson et al., 2025). The high level of enrollment and compliance in this sample due to the tailored-intervention approach supported the ability for this measurement approach that included multiple wearables and self-report measures of PA (Naqvi et al., 2024). This holistic approach to measuring activity has unique value during this period of PA research, wherein consumer wearables become more common and comparisons across these measurements are an important step for the field.

## Conclusions

The present analyses among Latina adolescents add to the existing body of literature that identifies reactivity to accelerometry, identifying changes to steps consistent with reactivity to ActiGraph wear; however, these differences are modest in the context of daily variability. The non-significant findings regarding types of PA suggest that ActiGraph wear may introduce minimal bias from reactivity, which validates a common PA measurement approach. Future research should consider a concurrent measurement approach as a method of triangulating PA levels to provide confidence in results and make findings comparable to the ways that consumers understand PA broadly.

## Figures and Tables

**Table 1.** Sample characteristics by wear day.

	ActiGraph Non-Wear Day	ActiGraph Wear Day	Overall
	(*N* = 1931)	(*N* = 1056)	(*N* = 2987)
**Valid Fitbit Wear Time (mins)**			
Mean (SD)	1210 (267)	1200 (267)	1210 (267)
**Timepoint**			
6-Month	1239 (64.2%)	696 (65.9%)	1935 (64.8%)
12-Month	692 (35.8%)	360 (34.1%)	1052 (35.2%)
**Day Type**			
Weekday	1378 (71.4%)	793 (75.1%)	2171 (72.7%)
Weekend	553 (28.6%)	263 (24.9%)	816 (27.3%)
**Age (yrs)**			
Mean (SD)	15.8 (1.67)	15.8 (1.61)	15.8 (1.65)
**Intervention Assignment**			
Treatment	931 (48.2%)	495 (46.9%)	1426 (47.7%)
Control	995 (51.5%)	559 (52.9%)	1554 (52.0%)
**Daily Steps**			
Mean (SD)	7330 (4190)	7620 (4220)	7430 (4200)
**MVPA Mins**			
Mean (SD)	29.1 (44.6)	29.7 (45.9)	29.3 (45.0)
**LPA Mins**			
Mean (SD)	237 (95.8)	242 (94.1)	239 (95.2)
**Sedentary Mins**			
Mean (SD)	1170 (114)	1170 (112)	1170 (113)

MVPA: Moderate to Vigorous Physical Activity, LPA: Light Physical Activity.

**Table 2.** Linear regression results by outcome^a^.

	Steps		MVPA		LPA		Sedentary	
	*Estimate (95% CI)*	*p-value*	*Estimate (95% CI)*	*p-value*	*Estimate (95% CI)*	*p-value*	*Estimate (95% CI)*	*p-value*
**ActiGraph Wear Day**	0.035	.0499	0.011	.785	4.006	.181	−5.254	.163
*vs Non-Wear Day*	(0.000, 0.070)		(−0.065, 0.086)		(−1.860, 9.872)		(−12.634, 2.127)	
**Valid Wear Time**	.162	<.001	0.032	.188	31.876	<.001	−31.236	<.001
*Continuous*	(0.102, 0.185)		(−0.016, 0.079)		(28.280, 35.472)		(−35.739, −26.733)	
**6-Month Timepoint**	−0.069	<.001	0.002	.958	−13.788	<.001	17.659	<.001
*vs 12-Month Timepoint*	(−0.109, −0.030)		(−0.083, 0.087)		(−20.302, −7.274)		(9.480, 25.839)	
**Weekend**	−0.170	<.001	0.103	.019	−3.069	.334	7.250	.0696
*vs Weekday*	(−0.208, −0.131)		(0.017, 0.191)		(−9.291, 3.154)		(−0.582, 15.081)	
**Age**	−0.013	.5649	0.008	.791	−1.041	.762	0.347	.9299
*Continuous*	(−0.556, 0.030)		(−0.050, 0.066)		(−7.780, 5.698)		(−7.380, 8.074)	
**Intervention Group**	0.058	.4201	0.013	.896	12.099	.279	−7.187	.5752
*vs Control Group*	(−0.082, 0.406)		(−0.176, 0.201)		(−9.808, 34.006)		(−32.318, 17.945)	

a All estimates are from the fully adjusted model.

MVPA: Moderate to Vigorous Physical Activity, LPA: Light Physical Activity.
